# Predictors of diffuse-type in-stent restenosis following drug-eluting stent implantation

**DOI:** 10.3892/etm.2013.1024

**Published:** 2013-03-21

**Authors:** CHANG-BUM PARK, HOON-KI PARK

**Affiliations:** 1 Department of Internal Medicine, Graduate School of Medicine, Kyung Hee University at Gangdong, Kyung Hee University, Seoul, Republic of Korea; 2 Department of Internal Medicine, Seoul Veterans Hospital, Seoul, Republic of Korea

**Keywords:** stent, restenosis, predictor

## Abstract

Diffuse-type in-stent restenosis (ISR) is known to be associated with a higher rate of restenosis than focal-type ISR. Therefore, it is clinically important to identify the determinants of diffuse-type ISR following drug-eluting stent (DES) implantation. We investigated the clinical, procedural and angiographic variables for predicting diffuse-type ISR following DES implantation. A total of 173 ISR lesions in 159 patients (diffuse-type: 61 lesions, focal-type: 112 lesions) following DES implantation from February 2003 to May 2008 were included in this study. Clinical, procedural and quantitative coronary angiographic variables were analyzed to determine predictors of diffuse-type ISR following DES implantation. Univariate analysis showed that the absence of hypertension [odds ratio (OR), 0.493; 95% confidence interval (CI), 1.025–4.103, P=0.042], use of a paclitaxel-eluting stent (PES) (OR, 3.318; 95% CI, 1.730–6.365, P<0.001) and smaller post-stenting minimal luminal diameter (MLD; OR, 0.368, 95% CI, 0.168–0.808, P=0.013) were significantly associated with diffuse-type ISR. However, use of a PES (OR, 3.957; 95% CI, 1.977–7.922, P<0.001) and smaller post-stenting MLD (OR, 0.320; CI, 0.140–0.731, P=0.007) were only independent predictors of diffuse-type ISR by multivariate analysis. Diabetes was not a predictor of diffuse-type ISR. The use of a PES and the post-stenting MLD were related to diffuse-type ISR following DES implantation.

## Introduction

Routine stent implantation has been shown to have a better procedural success rate and clinical outcome than balloon angioplasty (1,2). However, restenosis and repeat revascularization remain significant clinical problems limiting the long-term success of stent implantation (1–3).

Recent development of the drug-eluting stent (DES) has reduced the incidence of stent-related restenosis to <10%, but has not eliminated it completely (4,5). Furthermore, diffuse-type in-stent restenosis (ISR; ≥10 mm in length) has also been shown to be associated with a higher rate of recurrent restenosis and poor prognosis than focal-type ISR in the DES (6), as well as in the bare metal stent (BMS) (7–9). However, little data is available concerning predictors of diffuse-type ISR following DES implantation.

Therefore, the present study sought to identify the predictors of diffuse-type ISR following successful DES implantation.

## Patients and methods

### Patients

Between February 2003 and May 2008, 2,485 consecutive patients underwent coronary DES implantation in 3,466 lesions at Seoul Veterans Hospital (Seoul, Korea). Angiographic follow-up was obtained for 2,403 eligible lesions in 1,722 patients (follow-up rate, 69.3%), and the overall angiographic restenotic rate was 7.9% [189 lesions: sirolimus-eluting stent (SES), 107 lesions (56.6%); paclitaxel-eluting stent, (PES), 82 lesions (43.4%)]. Of these patients, 159 patients with 173 ISR lesions were enrolled in this study. Patients were excluded from this study if the treatment site was in the left main coronary artery (n=12) or venous bypass graft (n=4). This study was conducted in accordance with the Declaration of Helsinki and with approval from the institutional review board from the Kyung Hee University Hospital at Gangdong (Seoul, Korea).

### Stenting procedure

An SES (Cypher™; Cordis Corporation, Miami, FL, USA) or PES (Taxus™; Boston Scientific, Natric, MA, USA) was used in all patients. Stent implantation was performed according to standard techniques, and stents were selected at the physician’s discretion. Complete lesion coverage was recommended as well as angiographic optimization with <20% residual stenosis by visual estimation. During the procedure, patients received a bolus of 100 IU/kg heparin, with a repeated bolus of 2,000 IU heparin to maintain the activated clotting time of ≥300 sec. All patients were treated with aspirin 100–200 mg/day indefinitely and clopidogrel 75 mg/day for ≥6 months.

### Angiographic analysis

Coronary angiograms were analyzed by two experienced investigators who were not aware of the purpose of the study. The reference vessel diameter, the percent diameter stenosis and the minimal luminal diameter were determined using an online quantitative coronary angiographic system (Xelera Cath 1.1, Philips, The Netherlands) at baseline, subsequent to the procedure and again at follow-up. The angiographic measurement was made during the end-diastole following intracoronary nitroglycerin administration.

### Definitions

Demographic, clinical, angiographic and procedural characteristics were prospectively entered into the angiographic database of Seoul Veterans Hospital. Angiographic restenosis was defined as a diameter stenosis of ≥50% occurring in the segment inside the stent or a 5-mm segment proximal or distal to the stent at follow-up angiography. Patterns of restenosis were defined as follows; focal type: restenosis with a ≥50% luminal narrowing and <10 mm in length; and diffuse type: restenosis with a ≥50% lumimal narrowing and ≥10 mm in length.

### Statistical analysis

Data were expressed as the mean ± SD for continuous variables and frequencies for categorical variables. Continuous variables were compared by unpaired Student’s t-test and categorical variables by Chi-square test. Univariate regression analysis was performed on variables with a P-value of less than 0.05 to identify determinants of restenosis and variables found to be significant by univariate analysis were entered into multivariate analysis to determine their independent relationship to restenosis. A P-value of <0.05 was considered to indicate a statistically significant result.

## Results

Focal-type ISR developed in 112 lesions and diffuse-type ISR in 61 lesions. Follow-up angiography after coronary stenting was conducted a mean of 6.63±1.70 months after the procedure. Clinical, angiographic, and procedural variables were analyzed in this study and are summarized in Tables I and II. The diffuse-type ISR group had a lower incidence of absence of hypertension than the focal-type ISR group (37.3 vs. 54.6%, respectively, P=0.041), and a higher incidence of use of a PES (63.9 vs. 34.8%, respectively, P<0.001) and post-stenting minimal luminal diameter (MLD; 2.50±0.39 vs. 2.67±0.44 mm, respectively, P=0.011). However, diabetes was not significantly more common in diffuse-type ISR (19.6 vs. 28.7%, respectively, P=0.221).

Univariate analysis revealed that the absence of hypertension [odds ratio (OR), 0.493; 95% confidence interval (CI), 1.025–4.103, P=0.042], use of a PES (OR, 3.318; 95% CI, 1.730–6.365, P<0.001), and smaller post-stenting MLD (OR, 0.368; 95% CI, 0.168–0.808, P=0.013) were significantly associated with diffuse-type ISR. However, use of a PES (OR, 3.957; 95% CI, 1.977–7.922, P<0.001) and smaller post-stenting MLD (OR, 0.320; CI, 0.140–0.731, P=0.007) were only independent predictors of diffuse-type ISR by multivariate analysis (Table III).

## Discussion

This study showed that post-stenting MLD and the use of a PES are independent predictors of diffuse-type ISR. These results suggest that the largest possible post-stenting MLD and prudent selection of the type of DES are required to reduce the incidence of diffuse-type ISR.

Coronary stenting has become a standard therapy for coronary artery disease due to the simplicity of the procedure and its favorable outcomes. Recent randomized trials showed that the implantation of a DES dramatically reduced restenosis (4,5). However, restenosis continues to affect a significant number of patients with more complex lesions; therefore, more effective strategies are required.

Intravascular ultrasound has shown that ISR is the result of neointimal hyperplasia rather than elastic recoil (10,11). Mehran *et al*(9) developed an angiographic classification of ISR according to the geographic distribution of the intimal hyperplasia, demonstrating that repeat revascularization increases with increasing ISR class: pattern I (focal ISR, 19%), pattern II (diffuse ISR within the stent, 35%), pattern III (diffuse ISR outside the stent, 50%) and pattern IV (totally occluded ISR, 83%). Diffuse-type ISR reflects exaggerated neointimal hyperplasia as compared with the focal-type ISR and carries a higher rate of re-restenosis following BMS implantation and balloon angioplasty. Recently developed DESs have been shown to be effective in reducing binary restenosis in *de novo* lesions in randomized controlled trials by 0-9% (4,5). While this number is a great improvement over the results seen with BMS implantation, it is certainly not negligible. Furthermore, diffuse-type ISR following DES implantation is rare but continues to be associated with a higher rate of re-restenosis (6), as does BMS implantation (12,13). For these reasons, the identification of the predictors of diffuse-type ISR in DES implantation may play an important role in determining treatment strategies and prognosis.

In DES as well as BMS implantation, post-stenting MLD is one of the most powerful coronary morphologic predictors of restenosis among the many determinants of coronary morphology. Our data demonstrated that a smaller post-stenting MLD is associated with diffuse-type ISR. However, little is known concerning the relationship between diffuse-type ISR and post-stenting MLD. We suggest that a greater stent area may contribute to a decreased rate of diffuse-type ISR in patients with DES implantation but more investigative studies are required to confirm this hypothesis.

The current study also demonstrated that the use of a PES is associated with diffuse-type ISR. Other studies have reported similar results; the incidence of diffuse-type ISR following PES implantation has been reported to be 38–50% (14,15), compared with 0–14% following SES implantation (16,17). Furthermore, large clinical trials have also demonstrated that the SES is superior to the PES with regard to angiographic restenotic rate and late luminal loss (18,19). Whether this finding represents a more exaggerated neointimal response to the PES remains to be seen in the ongoing and the upcoming randomized trials between the two types of DES currently available on the market.

With DES, as with as BMS, diabetes continues to be an independent risk predictor of restenosis. Furthermore, a previous study (20) showed that diabetes was associated with diffuse-type ISR following BMS implantation. However, our results revealed that diabetes was not a predictor of restenosis following DES implantation, suggesting that the characteristics of ISR may be significantly different in the DES than in the BMS in diabetic patients but additional data are required to confirm this.

There were several potential limitations to the present study. Firstly, the choice of DES was made by the physician, which may have lead to a possible selection bias. Secondly, this study was a retrospective, single center study. Finally, our study was limited by the incomplete use of intravascular ultrasound. Despite these limitations, this study is based on one of the largest angiographic databases, and has identified independent predictors of diffuse-type ISR in DES implantation.

## Figures and Tables

**Table I:** Clinical characteristics of 159 study patients.

Characteristics	Diffuse-type ISR (n=51)	Focal-type ISR (n=108)	P-value
Age, years	58.0±12.4	59.0±9.9	0.602
Male gender	37 (72.5%)	76 (70.4%)	0.777
Diabetes	10 (19.6%)	31 (28.7%)	0.221
Hypertension	19 (37.3%)	59 (54.6%)	0.041
Current smoking	19 (37.3%)	32 (29.6%)	0.336
Hypercholesterolemia (≥200 mg/dl)	12 (23.5%)	28 (25.9%)	0.745
Prior PCI	11 (21.6%)	24 (22.0%)	0.926
Prior CABG	0 (0%)	1 (0.9%)	1.000
Clinical presentation			0.757
Stable angina	27 (52.9%)	65 (60.2%)	
Unstable angina	13 (25.5%)	21 (19.4%)	
NSTEMI	4 (7.8%)	10 (9.3%)	
STEMI	7 (13.7%)	12 (11.2%)	
LVEF (%)	59.2±8.8	58.3±8.5	0.540
Multivessel disease	28 (54.9%)	51 (47.2%)	0.336

Data are presented as mean ± SD or n (%). PCI, percutaneous coronary intervention; CABG, coronary artery bypass surgery; STEMI, ST-segment elevation myocardial infarction; NSTEMI, non-ST-segment elevation myocardial infarction; LVEF, left ventricular ejection fraction.

**Table II:** Procedural and angiographic variables for 173 study lesions.

Variables	Diffuse-type ISR (n=61)	Focal-type ISR (n=112)	P-value
Lesion characteristics			
Target coronary vessel			0.973
Left anterior descending	35 (57.4%)	66 (58.9%)	
Left circumflex	7 (11.5%)	13 (11.6%)	
Right coronary	19 (31.1%)	33 (29.5%)	
Type B2/C lesions	51 (83.6%)	102 (91.0%)	0.772
Chronic total occlusion	7 (11.5%)	12 (10.7%)	0.878
Restenotic lesion	6 (9.8%)	13 (11.6%)	0.722
Ostial lesion	2 (3.3%)	10 (8.9%)	0.162
Bifurcation	7 (11.5%)	18 (16.1%)	0.411
Procedural characteristics			
Balloon/artery ratio	1.26±0.18	1.28±0.19	0.568
Stent per lesion	1.66±0.87	1.73±0.74	0.572
Stent length per lesion, mm	42.2±25.1	45.0±23.7	0.453
Use of PES	39 (63.9%)	39 (34.8%)	<0.001
Quantitative coronary angiography			
Lesion length, mm	35.9±19.7	38.4±20.8	0.429
Reference vessel diameter, mm	2.71±0.53	2.77±0.43	0.448
Pre-intervention			
Minimal luminal diameter, mm	0.71±0.49	0.80±0.55	0.286
Diameter stenosis, %	72.3±17.4	70.5±19.6	0.544
Post-intervention			
Minimal luminal diameter, mm	2.50±0.39	2.67±0.44	0.011
Diameter stenosis, %	4.63±16.0	2.8±14.8	0.350
Postprocedural TIMI 3 flow	58 (95.1%)	111 (99.1%)	0.126
Acute gain	1.76±0.50	1.88±0.60	0.202
IVUS guidance	36 (59.0%)	81 (72.3%)	0.074

Data are presented as mean ± SD or n (%). PES, paclitaxel-eluting stent; TIMI 3, thrombolysis in myocardial infarction grade 3; IVUS, intravascular ultrasound.

**Table III:** Predictors of angiographic restenosis by logistic regression analysis.

Variables	Univariate analysis			Multivariate analysis		
	
	OR	95% CI	P-value	OR	95% CI	P-value
Use of PES	3.318	1.730–6.365	<0.001	3.957	1.977–7.922	<0.001
Post-stenting MLD	0.368	0.168–0.808	0.013	0.320	0.140–0.731	0.007
Hypertension	0.493	0.249–0.975	0.042	0.507	0.253–1.014	0.055

OR, odds ratio; CI, confidence interval; PES, paclitaxel-eluting stent; MLD, minimal luminal diameter.
